# Metastases to duodenum in cervical squamous cell carcinoma: A case report and review of the literature

**DOI:** 10.1097/MD.0000000000028526

**Published:** 2022-01-14

**Authors:** Yihan Chen, Hao Zhang, Qingjie Zhou, Lijie Lu, Jiejun Lin

**Affiliations:** Department of Gastroenterology, Wenzhou Central Hospital, Wenzhou, Zhejiang, China.

**Keywords:** case report, cervical squamous cell carcinoma, duodenal stenosis, duodenum, metastasis

## Abstract

**Rationale:**

Metastases to the duodenum in cervical squamous cell carcinoma are extremely rare, with only 7 cases reported in the published English literature.

**Patient concerns:**

We present the case of a 66-year-old woman with duodenal metastasis of cervical squamous cell carcinoma who presented with nausea and vomiting within the past 12 days.

**Diagnosis:**

Esophagogastroduodenoscopy revealed a circular narrowed 2nd part of the duodenum with congested and edematous mucosa, which was biopsied for a suspected neoplastic lesion. The pathological diagnosis indicated squamous cell carcinoma identical to the original tumor, confirming duodenal metastasis.

**Interventions:**

The patient received total parenteral nutrition on admission, but symptoms of jaundice soon appeared in the following week, suggesting infiltration of carcinoma into the common bile duct. After percutaneous transhepatic cholangial drainage was performed, jaundice eased in the following 3 days, and an uncovered self-expandable metallic stent was subsequently inserted into the stenosis of 2nd and 3rd part of the duodenum. Subsequently, the patient's diet quickly resumed.

**Outcomes:**

The patient refused further intervention and was discharged home to continue palliative care at the local hospital.

**Lessons:**

Clinicians should be alert to patients’ past medical history to ensure that duodenal metastasis of other tumors is considered in the differential diagnosis. For endoscopists, awareness of such patterns of duodenal stenosis is vital for the accurate recognition of such infrequent diseases.

## 1 Introduction

Compared with other sites, histopathological specimens of duodenal lesions are encountered relatively infrequently in endoscopic biopsies. Such lesions are rarely diagnosed as cancers, accounting for only 0.4% of all carcinomas.^[1]^ It is extremely rare to find squamous cell carcinoma in biopsy of the duodenal mucosa. Instead of squamous cell carcinoma, adenocarcinoma is the main primary cancer of the duodenum, and metastasis of other tumors accounts for 16.3% of duodenal malignancies.^[2]^ For squamous cell carcinoma, although lung cancer is the most common extra-gastrointestinal primary tumor to metastasize to the duodenum, metastases from other tumors have occasionally been reported.

Cervical cancer is the fourth most common gynecologic malignancy worldwide, and the majority of cases are attributed to human papillomavirus infection.^[3]^ The primary mechanisms of cervical cancer metastasis include direct local extension and lymphatic dissemination, while hematogenous dissemination rarely occurs.^[4]^ Common distant metastatic sites include the lungs, bones, and liver, whereas cervical cancer metastasis to the duodenum is infrequently encountered.

In view of the rarity of cervical cancer with duodenal metastasis, we herein present a case of a cervical cancer patient with duodenal metastases confirmed by histopathological diagnosis presenting with obstruction symptoms.

## 2 Case presentation

A 66-year-old woman was admitted to our department with a 12-day history of progressive nausea and vomiting of the gastric content. The patient's vital signs were stable on physical examination and no lesions were found in the oropharynx or nasopharynx. She had light abdominal distension, and there was no abdominal tenderness or rebound tenderness. A careful past history was taken on admission that she had undergone radical hysterectomy with adnexectomy and radiation treatment for invasive squamous cell carcinoma (stage IB) of the cervix 9 years prior. The postoperative pathology analysis showed that the removed tissue was cervical low-differentiated squamous cell carcinoma with a maximum diameter of 27 mm and infiltration into 1/2 of the cervical stroma. No lymph node metastases were found in the bilateral pelvic or common iliac lymph nodes. After the operation, the patient was followed up at a local hospital for a long time and received radiotherapy.

**Figure 1:**
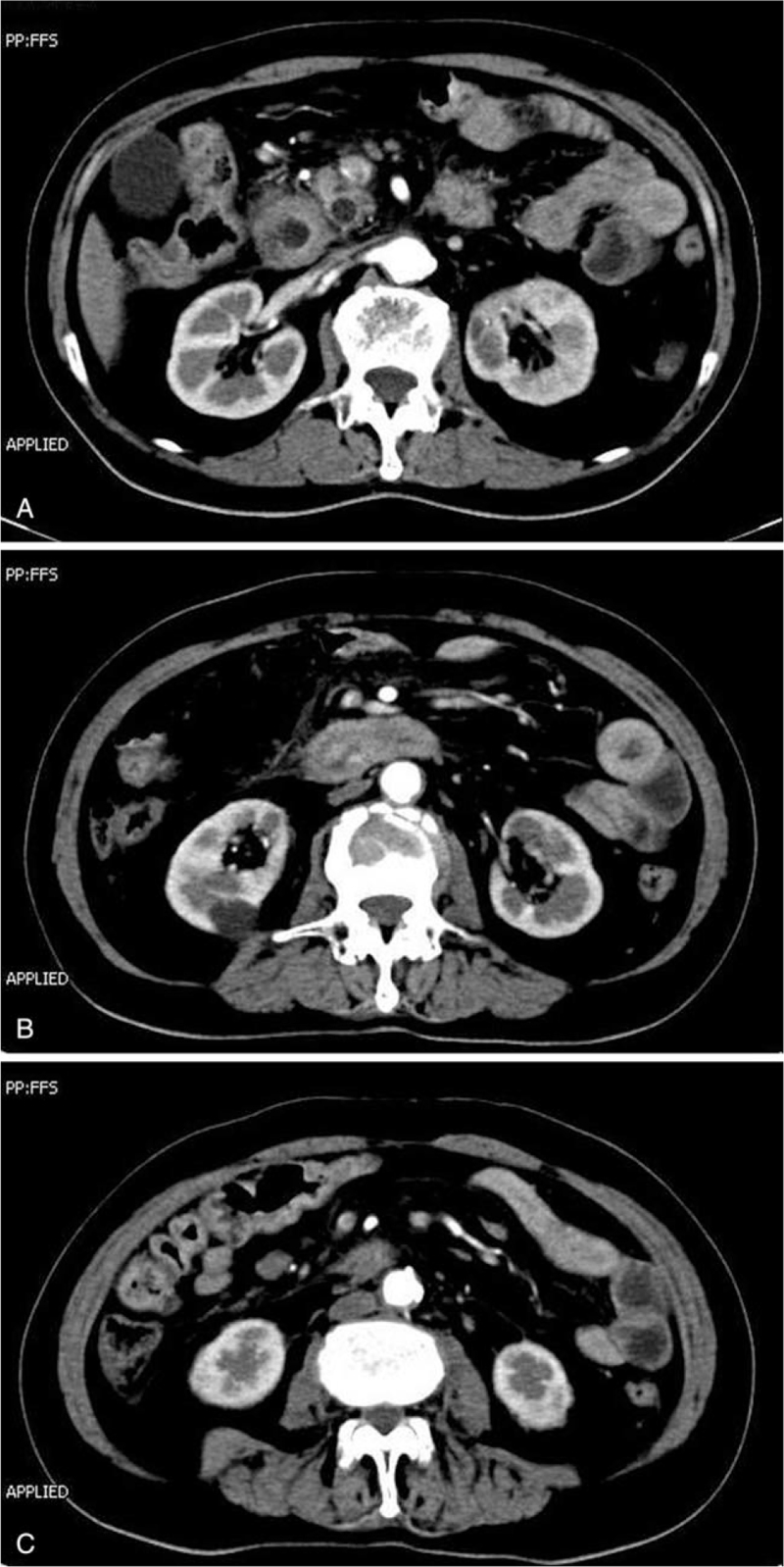
Abdominal contrast enhanced computerized tomography. A: CECT showed stenosis of the lower end of the common bile duct and a thickened bowel wall of 2nd part of duodenum with exudation around. B: CECT demonstrated extensive edema and thickening of the intestinal wall in 3rd part of the duodenum. C: CECT showed multiple enlarged lymph nodes in the retroperitoneal space and mesenteric space. CECT = contrast enhanced computerized tomography.

**Figure 2:**
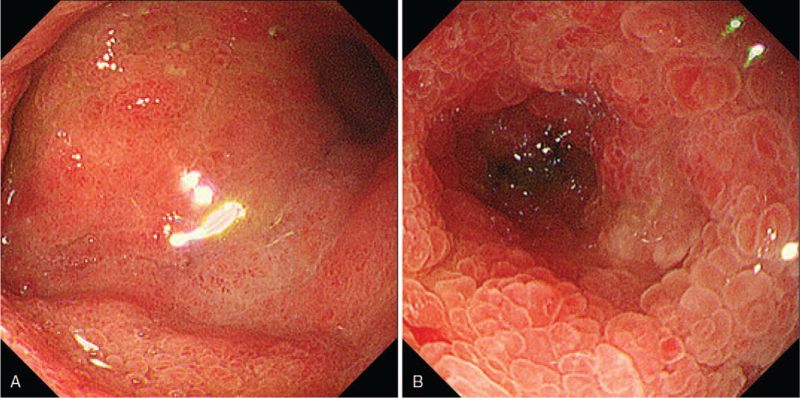
Esophagogastroduodenoscopy. A: Esophagogastroduodenoscopy confirmed that the mucosa of 1st part of duodenum was smooth without ulcers or lumps. B: Esophagogastroduodenoscopy showed congested and edematous mucosa seen in circle wall of 2nd part of duodenum with almost complete luminal obstruction that transnasal gastroscope could not pass through.

**Figure 3:**
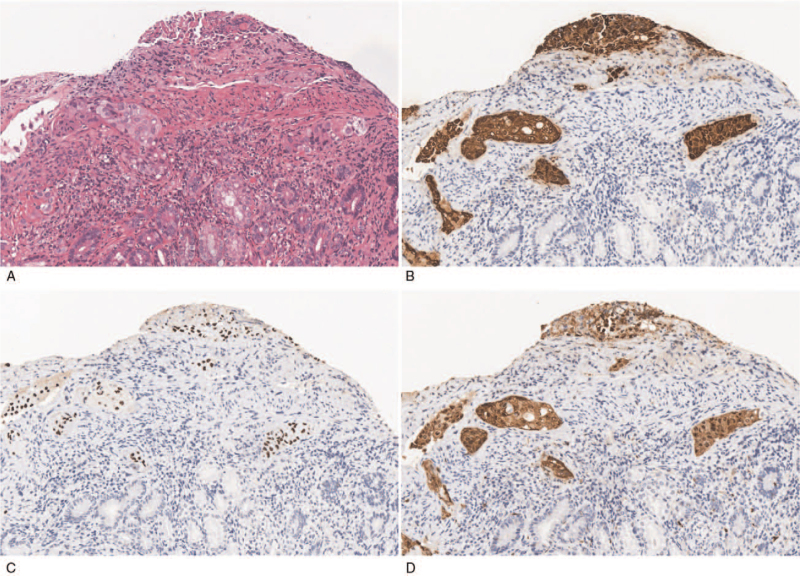
Duodenal biopsy. A: Photomicrograph of haematoxylin and eosin stained slide showed the presence of malignant nonkeratinizing squamous cells arranged in clumps and nests which adjacent to normal duodenal mucosa. B: Photomicrograph of staining with Cytokeratin 5/6 shows strong cytoplasmic and membrane staining of the diseased cells with no staining in the adjacent duodenal mucosa. C: Photomicrograph of staining with P63 shows nuclear staining of the diseased cells with no staining of adjacent duodenal mucosa. D: Photomicrograph of staining with P16 shows diffuse cytoplasmic and nuclear staining of the diseased cells with no staining in the adjacent duodenal mucosa (×400 magnification).

The relevant examinations were completed immediately after admission. Abdominal contrast-enhanced computerized tomography confirmed stenosis of the lower end of the common bile duct and a thickened bowel wall of 2nd and 3rd part of the duodenum with surrounding exudation, as seen in Fig. 1A and B. Figure 1C shows multiple enlarged lymph nodes in the retroperitoneal and mesenteric spaces, suggesting disseminated carcinomatosis. Esophagogastroduodenoscopy revealed a circular narrowed 2nd part of the duodenum with congested and edematous mucosa that the transnasal gastroscope could not pass through (Fig. 2A and B). We then obtained a tissue biopsy of the mucosa of the 2nd part of the duodenum, and pathological results indicated the presence of neoplastic cells arranged in clumps and nests adjacent to the normal duodenal mucosa, which is highly reminiscent of malignant squamous cell carcinoma (Fig. 3A). In this context, immunohistochemical staining indicated that the diseased cells stained positive for antibodies against CK5/6, p63, and p16 (Fig. 3B–D). A good internal control of negative staining in the adjacent duodenal mucosa was observed.

**Figure 4:**
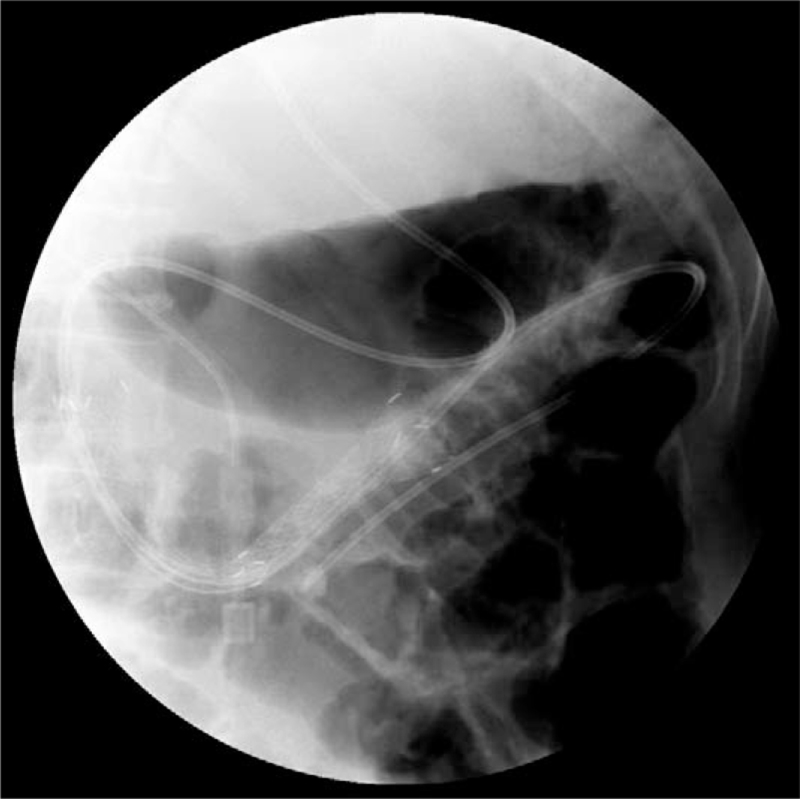
Abdominal radiograph showed that the 10-cm stent started in the 2nd part of the duodenum and ended in the 3rd part of the duodenum. The length of duodenal stenosis is nearly 7 cm and the diameter of the narrowest part was about 2 mm.

The patient fasted and received total parenteral nutrition on admission, but symptoms of jaundice soon appeared in the following week, suggesting infiltration of squamous cell carcinoma into the lower end of the common bile duct. Considering the patient's condition, imaging findings, and esophagogastroduodenoscopy results, percutaneous transhepatic cholangial drainage was performed. Jaundice eased in the following 3 days, and an uncovered self-expandable metallic stent (100 mm length, 20 mm diameter, BONASTENT, Sewoon Medical, Korea) was subsequently inserted in the stenosis of 2nd and 3rd part of the duodenum that the patient's diet was quickly resumed (Fig. 4). However, the patient refused further intervention and was discharged home to continue palliative care at the local hospital.

## 3 Discussion

Squamous cell carcinoma (SCC) of the cervix accounts for 75% to 90% of all cases of cervical cancer and is the fourth most common cancer among women, with 10% to 25% being adenocarcinomas.^[5]^ The global age-related incidence of cervical cancer is estimated to be 13.1/100,000, which varies widely among countries. Due to the different economic levels and medical resources of various countries, cervical cancer incidence has been declining in Western, developed countries, mainly as a result of the promotion of human papillomavirus vaccines, while low-income and developing countries have experienced increasing incidence and mortality of cervical carcinoma. On average, the age at diagnosis of cervical cancer is 53 years and the age at death is 59 years worldwide.^[6]^ The stage and prognosis of cancer are closely related; nearly 60% of cases are identified at stage I, with 25%, 10%, and 5% detected in stages II, III, and IV, respectively.^[7]^ Cervical cancer mainly spreads in an orderly and predictable manner, directly extending to adjacent structures, such as the vagina, paracervical tissue, bladder, ureter, and rectum, while distant metastatic spreads are unpredictable. The actuarial incidence of 10-year distant metastases is 3% in stage IA, 16% in stage IB, 31% in stage IIA, 26% in stage IIB, 39% in stage III, and 75% in stage IVA.^[8]^ After precise calculation, the proportions of lung, liver, bone, and brain distant metastases were identified in 59%, 16%, 23%, and 2% of patients with cervical cancer, respectively.^[9]^ Gastrointestinal tract metastases can be found in approximately 8% of cervical cancer patients; these are common in the rectum and sigmoid colon as a result of direct local extension, whereas cervical cancer metastasis to the duodenum is infrequently encountered.

Cervical cancer metastasis to the duodenum is rare. A comprehensive review of published cases of duodenal metastases from SCC of the cervix in the English literature dating back to 1981 is provided in Table 1.^[10–16]^ As shown in Table 1, since 1981, even with our report, only 8 cases of duodenal metastasis of cervical cancer have been reported. It is worth noting that the interval time to metastases in our cases was significantly higher than in the others, which may be due to the earlier stage of SCC of the cervix or the suppression of tumor cells by the previous treatment of hysterectomy and radiation therapy. Endoscopy is an extremely important diagnostic method, and owing to the development of endoscopy in recent years, most cases have been reported in the last 10 years. This did not go the way we thought because not only do cervical cancer metastases to the duodenum mainly occur at stage IV, but they also occur at an early stage, even at stage IB. Unfortunately, a duodenal metastatic tumor indicates a poor prognosis, and the outcome of most patients is either death or loss to follow-up. For example, jaundice soon appeared in our patients because of rapid cancer progression within a few days after admission, suggesting the infiltration of cancer cells in the common bile duct, which shows the significance of a fast and accurate diagnosis.

**Table 1:** Duodenum metastases from cervical cancer as reported in the literature.

No.	Authors	Year	Age	Stage of SCC of cervix at diagnosis	Previous treatment	Time interval to metastases	Presenting symptoms	Metastasis sites	Confirmation of diagnosis	Follow-up treatment	Outcome
1	Gurian L	1981	64	IIIb	None	Synchronous metastases	Occult bleeding	1st part of duodenum	Endoscopy	Refused surgical intervention	Death
2	Kanthan R	2011	49	IIA	Chemotherapy and radiation treatment	2 years	Upper gastrointestinal bleeding	2nd part of duodenum	Endoscopy	None	Death
3	Lee TH	2011	50	IIA	Hysterectomy	2 years	Epigastric pain	2nd part of duodenum	Endoscopy	Chemotherapy	NA
4	Raphael JC	2011	57	IV	None	Synchronous metastases	Persistent epigastric pain and vomiting	1st part of duodenum	Endoscopy	Chemotherapy	NA
5	Chawhan SM	2015	52	NA	Hysterectomy and radiation therapy	2 years	Abdominal pain associated with heartburn and nausea.	3rd part of duodenum	Endoscopy	NA	NA
6	Subramanian K	2016	50	IIA	Radiation therapy	2 years	Upper abdominal pain, loss of appetite, and weight of 1 month duration.	1st part of duodenum	Endoscopy	Chemotherapy	Recovery
7	Ash J	2021	81	IVA	Radiation therapy	3 years	Severe abdominal pain	1st and 2nd part of duodenum	Endoscopy	Refused further interventions	Death
8	Chen YH	2021	66	IB	Hysterectomy and radiation therapy	9 years	Nausea and vomiting	2nd and 3rd part of duodenum	Endoscopy	Refused further interventions	None

NA = not available from original literature, SCC = squamous cell carcinoma.

When duodenal stenosis is found during endoscopy examination, as an endoscopist, consideration of the most possible diagnosis and differential diagnosis is necessary. There are 2 types of duodenal stenosis: congenital and acquired. Congenital duodenal stenosis is usually diagnosed 4 days to 1 month after birth, with an incidence of 1 in 5000 to 1 in 10,000 live births.^[17]^ Approximately half of these patients are premature, while adult patients are occasionally reported, with a fixed narrowed pylorus with a smooth border as the classic finding on endoscopy.^[18]^ There are several causes of acquired duodenal stenosis: postbulbar duodenal ulceration, related to *Helicobacter pylori* infection, is the most common cause of duodenal stenosis.^[19]^ Endoscopy often shows ulcers and irregular scar formation, presenting with eccentric stenosis of the duodenum with background mucosa of the stomach, indicating *H pylori* infection. As an autoimmune disease that can occur in the entire gastrointestinal tract, duodenal involvement represents 3.6% of Crohn disease patients.^[20]^ On endoscopic examination, many patients present with a friable granular mucosa and multiple superficial erosions or aphthoid ulcers. Duodenal tuberculosis is a rare clinical entity. Luminal stenosis may be the only endoscopic finding, and endoscopic biopsy findings are nonspecific. The lack of special manifestations leads to a final diagnosis that requires surgical intervention.^[21]^ Primary duodenal adenocarcinoma accounts for nearly half of all small-bowel adenocarcinomas. While no tumors arise from 1st part of the duodenum, 87% of duodenal adenocarcinoma occurs on 2nd part of the duodenum.^[22]^ The endoscopic characteristics of the lesions include irregular huge ulcers covered with necrotic tissue, irregular patchy erosion of mucosa with absent peristalsis, and cauliflower-like mass with superficial ulcer. The use of magnifying endoscopy might help to detect cancer. However, almost 16.3% of carcinomas were reclassified as metastatic lesions arising from other tumors, which requires a careful history to be taken as tips for the correct diagnosis. Stenosis of the duodenum due to compression by other tissues, such as ruptured pancreaticoduodenal artery aneurysms, is characterized by luminal narrowing without visible mucosal lesions on endoscopy.^[23]^ The involvement of inflammation in the duodenum could cause congestion and edema of the intestinal wall, resulting in lumen stenosis. For example, an inflammatory reaction and fluid collection caused by acute pancreatitis dissect into the groove between the pancreatic head and descending duodenum, which may lead to duodenal stenosis.^[24]^ Occasionally, it has been reported that some special pathogen infections, such as strongyloidiasis, can also cause duodenal stenosis.^[25]^

In general, metastases to the duodenum in cervical squamous cell carcinoma are extremely rare, and only 7 cases have been reported in the published English literature worldwide. Symptoms of obstruction such as abdominal pain, nausea, and vomiting are common clinical features. As the tumor progresses, infiltration of the common bile duct leads to obstructive jaundice. Duodenal metastasis from cervical cancer can be effectively diagnosed using endoscopy and pathological biopsy. Owing to the lack of sufficient cases to compare the efficacy of different treatments, the best treatment remains controversial. Laparotomy is a good choice for physically capable patients. However, the poor prognosis and rapid progression of this disease have resulted in no patient being capable of receiving surgical treatment; most patients finally refused further interventions, and chemotherapy could only be employed as a palliative treatment.

## 4 Conclusions

In conclusion, this report presents an extremely rare case of cervical cancer metastasis to the duodenum leading to obstruction. Clinicians should be alert to patients’ past medical history to ensure that duodenal metastasis of other tumors is considered in the differential diagnosis. For endoscopists, awareness of such patterns of duodenal stenosis is vital for the accurate recognition of such infrequent diseases.

## Author contributions

**Investigation:** Qingjie Zhou.

**Writing – original draft:** Yihan Chen, Hao Zhang.

**Writing – review & editing:** Lijie Lu, Jiejun Lin.
